# Dataset of molecular dual-atom site complexes for catalysis

**DOI:** 10.1016/j.dib.2026.112806

**Published:** 2026-05-01

**Authors:** Ba Long Nguyen, Thor Kongstad Madsen, Vladislav Ivanistsev, Nadezda Kongi

**Affiliations:** a Institute of Chemistry, University of Tartu, Ravila 14a, 50411 Tartu, Estonia; b Department of Chemistry, University of Latvia, Jelgavas iela 1, 1004 Riga, Latvia

**Keywords:** Dual-atom site molecular catalysts, Computational catalysis, Large-scale catalyst database, High-throughput screening, Density functional

## Abstract

Dual-atom site catalysts (DACs) are characterized by two adjacent metal centers embedded within a well-defined coordination environment. The close proximity of the metal sites enables cooperative interactions, allowing more flexible tuning of adsorption energetics and reaction pathways. To accelerate rational design, screening, and comparative analysis of DACs, standardized, reusable computational datasets are essential. In this work, we present a dataset of molecular DACs (i.e., complexes) in a human- and machine-readable Atomic Simulation Environment (ASE)-native JSON format. The data were generated using an automated computational workflow, which has been made available on Zenodo. Geometry optimizations and single-point calculations were performed using density functional theory. For each catalyst, the optimized structure is provided together with single-point geometric, energetic, and electronic properties.

Specifications Table

**Table:** 

Subject	Chemistry
Specific subject area	Computational Catalysis
Type of data	Atomic Simulation Environment (ASE)-native JSON
Data collection	High-throughput automated workflow with structure generation, geometry optimization, single-point calculation, and direct database storage
Computational method	The spin-polarized GFN2-xTB (spGFN2-xTB) method was used to generate the dataset version 1.0
Parameters for data collection	Total charge and spin multiplicity, spin polarization, solvation, maximum number of iterations, force convergence threshold
Data source location	University of Tartu and University of Latvia
Data accessibility	Repository name: Dataset of molecular Dual-Atom Site complexes for catalysis Data identification number: 10.5281/zenodo.18601379 Direct URL to data: https://zenodo.org/records/18601379 Instructions for accessing these data: The dataset is openly available on Zenodo at the DOI listed above. All data files can be accessed through the provided URL and downloaded individually or as a compressed archive. The repository includes a human- and machine-readable database file (in ASE-native JSON format), along with metadata, a YAML schema describing all fields, and documentation explaining the dataset structure and usage.
Related research article	None

## 1 Value of the Data

• The dataset provides a pool of organic dual-atom site molecular catalyst structures, benefiting researchers in computational catalysis, materials discovery, and data-driven catalyst design, particularly those exploring this new family of catalysts.
• The dataset is provided in an Atomic Simulation Environment (ASE)-native JSON format, enabling straightforward querying, filtering, and reuse within the ASE, while remaining fully human-readable.
• The dataset can be used to construct structure-property relationships; for example, by correlating computed adsorption energies using higher-level density functional theory (DFT) calculations with the available frontier orbital energies, atomic charges, and atom-resolved spin properties.

## 2 Background

Single-atom site catalysts (SACs) have been widely applied in electrochemical energy-conversion reactions, including the oxygen evolution reaction (OER), oxygen reduction reaction (ORR), and carbon dioxide reduction reaction (CO_2_RR) [[1], [2], [3]], their intrinsic limitations have been shown in multiple studies:

– First, the presence of a single isolated active site imposes strong scaling relations among reaction intermediates, limiting independent optimization of energetics across multi-step reaction pathways [4,5].
– Secondly, the lack of continuous or cooperative active sites restricts reaction-pathway diversity, resulting in poor selectivity toward target products [6,7].
– Third, maintaining isolated single-atom configurations requires very low metal loadings, which limits the achievable density of active sites and constrains the overall catalytic performance [8,9].

In this context, dual-atom site catalysts (DACs), as an extension of SACs, have been proposed to address these challenges. Cooperative interactions between adjacent metal centers have been shown to modulate adsorption energetics, break scaling relations inherent to single-atom sites, and enable high catalytic activity and stability in both molecular [10] and periodic binuclear coordination environments [11]. In this work, we focus on molecular DACs, for which publicly available, standardized datasets remain scarce.

First-principle approaches based on DFT [12,13] are the minimum standard for calculations. However, their application in large-scale catalyst screenings can be limited by their high resource requirements. In this case, a poor man’s approach is to use semi-empirical methods derived from the DFT, such as density functional tight-binding (DFTB) and related extended tight-binding approaches [[14], [15], [16], [17], [18]]. They provide a practical compromise by substantially reducing computational cost while retaining a consistent geometric- and electronic-structure description. In particular, the GFN2-xTB method [16] has been established as a robust and reliable tool for molecular structure optimization for metal-organic systems [19,20]. The method has been extended to include spin-dependent energy terms, enabling more accurate spin-polarized calculations, and is referred to as spGFN2-xTB [18]. Building on this balance between accuracy and efficiency, we present a large and reproducible dataset of molecular DACs, generated using the spGFN2-xTB method within an automated computational workflow.

## 3 Data Description

The dataset version 1.0 contains 44,190 molecular DACs, provided as a single ASE-native JSON file. Each entry contains the optimized catalyst geometry, along with the default structural and chemical information required to reconstruct the system in the ASE. In addition, geometric, energetic, and electronic properties obtained from single-point calculations, including atom-resolved properties derived via xTBML post-processing [21], are included. Associated metadata records of the identities of the metal centers and ligand fragments, the total molecular charge and spin multiplicity, and the optimization and single-point calculation settings are included, ensuring full reproducibility. Catalyst identifiers follow the naming convention described below.

The additional data columns beyond those provided by default in the ASE are described in Table 1 and Supplementary SI1.

**Table 1:** Columns describing molecular metadata, geometry optimization, and calculation settings, and core single-point calculation results, as stored under the *key_value_pairs* column in the ASE-native JSON representation of each structure. Columns prefixed with *SP_* correspond to quantities obtained from the single-point electronic-structure calculations.

Column key	Description
**Metadata and calculation settings**	
Name	Unique identifier of the molecular catalyst
metal1	Element symbol of the first metal center
metal2	Element symbol of the second metal center
Acid	Acid fragment identifier used in catalyst construction
Base	Base fragment identifier used in catalyst construction
Bridge	Identifier of metal-sharing bridges, if present
total_charge	Total molecular charge of the catalyst
multiplicity	Spin multiplicity used in geometry optimization and single-point calculations
opt_tblite_settings	Serialized dictionary of parameters used for spGFN2 geometry optimization via tblite, stored as a JSON string
sp_calc_settings	Serialized dictionary of parameters used for spGFN2 single-point calculations, stored as a JSON string
**Core single-point calculation results**	
SP_energy_eV	Total electronic energy obtained from the single-point calculation, in eV
SP_mulliken_charge	Atom-resolved Mulliken charges from the single-point calculation, stored as a JSON-serialized array in *e*
SP_orbital_energy_eV	Orbital energy levels from the single-point calculation, stored as a JSON-serialized array in eV
SP_orbital_occupation	Orbital occupation numbers from the single-point calculation, stored as a JSON-serialized array
SP_HOMO_eV	HOMO energy derived from SP_orbital_energy_eV and SP_orbital_occupation, in eV
SP_LUMO_eV	LUMO energy derived from SP_orbital_energy_eV and SP_orbital_occupation, in eV
SP_HOMO_LUMO_gap_eV	HOMO-LUMO energy gap in eV
SP_spin_per_atom	Atom-resolved spin populations computed from xTBML post-processing properties, stored as a JSON-serialized array

Table 1 lists molecular metadata, geometry optimization and calculation settings, and core single-point calculation results, whereas Table A.2 describes xTBML-derived properties.

All the additional columns are stored under the *key_value_pairs* column and are automatically mapped to individual database columns when the JSON representation is imported as an ASE SQLite database.

## 4 Computational Design

**Fig. 1:**
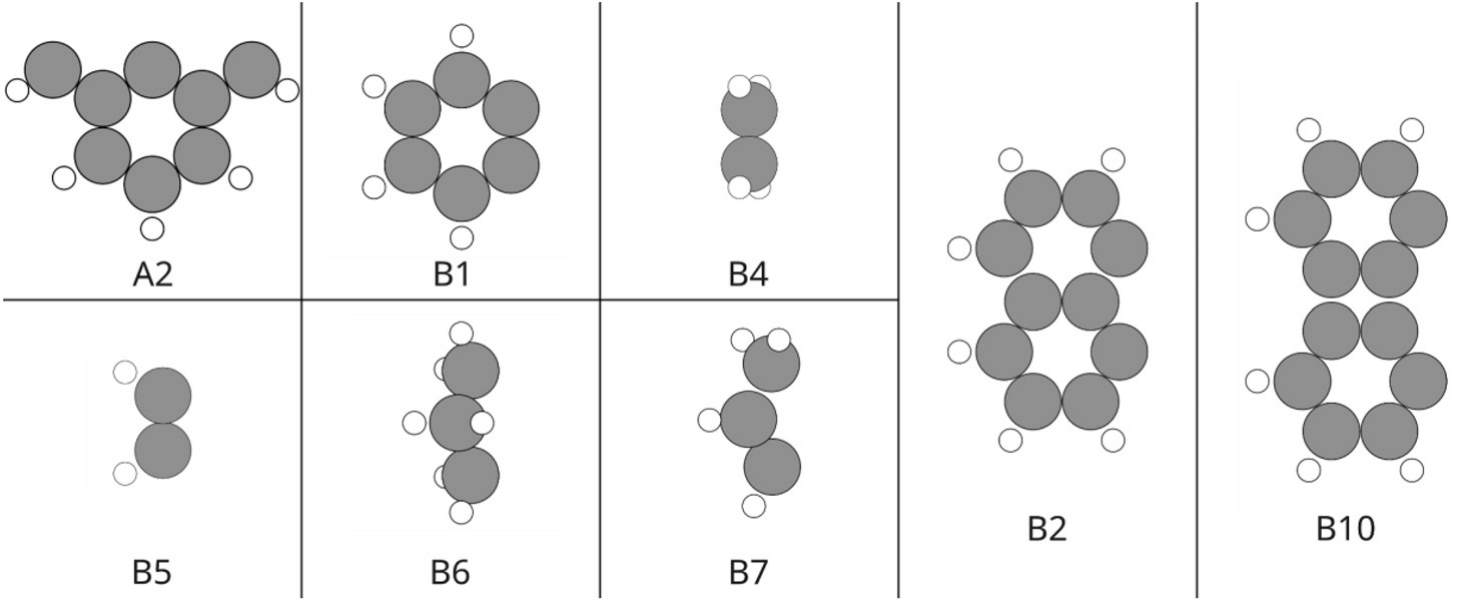
Acid (A2) and base fragments (B1, B2, B4, B5, B6, B7, B10) used for catalyst structure generation. Gray and white circles represent carbon and hydrogen atoms, respectively. The fragments are intentionally defined with bond-deficient carbon atoms to represent open valence sites, which become saturated through bond formation with other fragments.
• Base fragments: predefined organic fragments containing two single-bond-deficient carbon sites, as shown in Fig. 1.
• Bridges: atoms shared between acid fragments and the two metal centers, selected from {N, O, S}.
• Four nitrogen atoms form the acid–base linking frame.

**Fig. 2:**
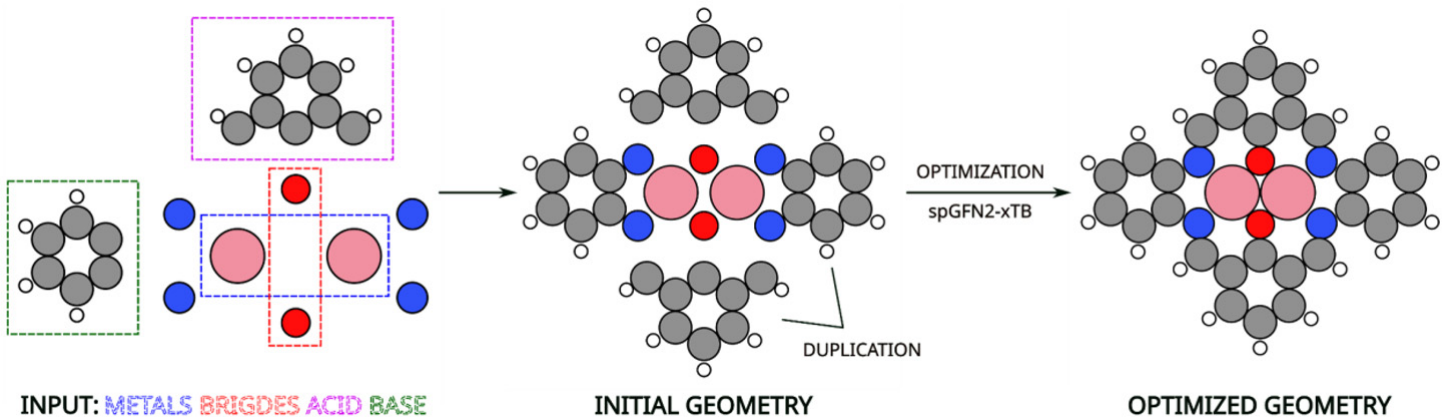
Schematic illustration of the generation of the KMC_Co_Co_A2_B1_NNNNOO_q2 catalyst: input selection (metals, bridges, acid, and base), initial geometry construction, and geometry optimization. Pink, red, blue, gray, and white circles represent cobalt, oxygen, nitrogen, carbon, and hydrogen atoms, respectively.

Input structures were generated using an automated Python-based workflow utilizing the ASE [22], v3.26.0. Initial structures were generated from a common template-based assembly procedure using predefined site, acid, and base fragments, with total charge systematically assigned based on the metal centers and bridge atoms, as illustrated in Fig. 2:

• Metal centers: two metal atoms selected from a predefined element set, including an s-block metal (Mg), 3d transition metals (*Sc*, Ti, V, Cr, Mn, Fe, Co, Ni, Cu, Zn), 4d transition metals (Y, Zr, Nb, Mo, Ru, Rh, Pd, Ag, Cd), 5d transition metals (Hf, Ta, W, *Re*, Os, Ir, Pt, Au, Hg), and selected p-block metals (Ga, In, Sn, Pb, Bi), occupying two equivalent metal sites.
• Acid fragments: predefined organic fragments containing two double-bond-deficient carbon sites and one single-bond-deficient carbon site, as shown in Fig. 1.

This ensures a standardized and reproducible starting point across the dataset, which is advantageous for high-throughput generation.

The connectivity between these fragments is defined through predefined valence deficiencies. Each single-bond-deficient carbon site on the base fragment forms a single covalent C–N bond with a nearby linking nitrogen atom. Simultaneously, the double-bond-deficient carbon sites on the acid fragment form covalent C = N double bonds to the same linking nitrogen atoms. This construction yields a fully connected organic frame. This mimics condensation reactions between aldehyde precursors and amine precursors to form imine linkages.

Each catalyst was assigned a unique name that encodes its composition. The identifier includes the two metal elements, the acid and base fragment labels, the four nitrogen atoms that link the organic frame (“NNNN”), the bridge's identity, and the total molecular charge. For example, *KMC_Co_Co_A2_B1_NNNNOO_q2* denotes a catalyst with two cobalt centers, acid fragment A2, base fragment B1, oxygen bridges, and a total molecular charge of +2. This naming convention ensures a one-to-one correspondence between the identifier, the molecular structure, and the database entry.

Fixed-*xy*-plane constraints were applied to all non-hydrogen, non-metal atoms that constitute the organic frame of the molecules. Geometry optimizations were performed using the spGFN2-xTB method [16,18] as implemented in the TBLite interface (v0.5.0), employing the BFGS algorithm [23]. Spin configurations were automatically tested, ranging from the lowest possible spin state to three successive higher spin states. The initial multiplicity was determined from the total electron count, and subsequent multiplicities were generated by increasing the multiplicity in steps of two. Geometry optimization was attempted sequentially for each multiplicity, and the first to converge was retained. Structures for which none of the tested multiplicities converged were recorded as failed. Out of 47,628 generated structures, 44,190 were successfully optimized, corresponding to a success rate of approximately 92.8%, while 3438 structures (7.2%) did not converge. The final multiplicity distribution spans singlet to octet states, with singlet (24,743) and doublet (5171) states accounting for the majority of the dataset (approximately 68%). Intermediate- and higher-spin states, including triplet (2136), quartet (8493), quintet (264), sextet (2821), septet (70), and octet (492), are also observed. Failed optimizations were primarily associated with SCF convergence issues, and no clear systematic trends with respect to metal combinations, ligand fragments, or charge states were identified.

A schematic illustration of fragment selection, initial geometry construction, and geometry optimization is shown in Fig. 2.

Single-point calculations using the same spGFN2-xTB method were performed on the optimized geometries, with xTBML post-processing enabled. All results and associated metadata were written to an ASE database. The ASE database was then converted to the ASE-native JSON format to ensure human readability.

## Limitations

The dataset version 1.0 is restricted to the chemical space defined in the current workflow setup, including a predefined set of metals, selected acid and base fragments, and bridge atoms limited to N, O, and S. Although the workflow is modular and can be extended to include additional metals, fragments, or alternative bridge chemistries such as B- or P-containing motifs, such extensions are not included in the current dataset. Also, the use of a common initial construction strategy may bias optimization toward local minima connected to that starting arrangement. The dataset is therefore intended as a standardized and internally consistent screening-level resource for high-throughput analysis, rather than an exhaustive representation of all possible chemistries or conformational space. In addition, comprehensive validation against higher-level DFT calculations is not included and is the subject of ongoing work, focusing on representative subsets and the correlation of the present geometric and energetic descriptors with more accurate electronic-structure methods.

## Ethics Statement

The authors have read and followed the ethical requirements for publication in Data in Brief and confirmed that the current work does not involve human subjects, animal experiments, or any data collected from social media platforms.

## CRediT Author Statement

**Ba Long Nguyen:** Methodology, Software, Investigation, Data curation, Formal analysis, Visualization, Writing – original draft, Writing – review & editing; **Thor Kongstad Madsen:** Methodology, Software, Writing – review & editing, Supervision; **Vladislav Ivanistsev:** Conceptualization, Methodology, Software, Writing – review & editing, Supervision, Project administration; **Nadezda Kongi:** Conceptualization, Supervision, Project administration.

## Data Availability

zenodoDataset of molecular Dual-Atom Site complexes for catalysis (Original data).

## Appendix Supplementary materials

Image, application 1
