# Supplementary material for: Dataset of molecular dual-atom site complexes for catalysis
Source: Data Brief. 2026 May 1;66:112806. doi: 10.1016/j.dib.2026.112806 (PMC13191655; doi:10.1016/j.dib.2026.112806)
Supplement: Supplementary file 1 [file mmc1.docx]

# xTBML-derived properties

| **Column key** | **Description** |
| --- | --- |
| **Geometry-based properties** | |
| SP_xtbml_CN_A | D3 coordination number |
| SP_xtbml_ext_CN | Extended coordination number obtained via the xTBML convolution kernel |
| **Energy-based properties** | |
| SP_xtbml_E_rep | Repulsion energy contribution, in Hartree |
| SP_xtbml_E_ies_ixc | Isotropic electrostatic and exchange–correlation energy contribution, in Hartree |
| SP_xtbml_E_axc | Anisotropic exchange–correlation energy contribution, in Hartree |
| SP_xtbml_E_aes | Anisotropic electrostatic energy contribution, in Hartree |
| SP_xtbml_E_disp2 | Two-body dispersion energy contribution, in Hartree |
| SP_xtbml_E_disp3 | Three-body dispersion energy contribution, in Hartree |
| SP_xtbml_E_eht | Extended Hückel energy contribution, in Hartree |
| SP_xtbml_E_tot | Total energy contribution obtained by summing the listed energy terms, in Hartree |
| SP_xtbml_w_tot | Weight in the total energy, defined as w_A,tot_ = E_A,tot_ / E_tot_ |
| **Orbital energy-based properties** | |
| SP_xtbml_HOAO_alpha | HOAO energy for the alpha spin channel, in eV |
| SP_xtbml_HOAO_beta | HOAO energy for the beta spin channel, in eV |
| SP_xtbml_LUAO_alpha | LUAO energy for the alpha spin channel, in eV |
| SP_xtbml_LUAO_beta | LUAO energy for the beta spin channel, in eV |
| SP_xtbml_gap_alpha | HOAO–LUAO energy gap for the alpha spin channel, in eV |
| SP_xtbml_gap_beta | HOAO–LUAO energy gap for the beta spin channel, in eV |
| SP_xtbml_chem_pot_alpha | Chemical potential for the alpha spin channel, in eV |
| SP_xtbml_chem_pot_beta | Chemical potential for the beta spin channel, in eV |
| SP_xtbml_response_alpha | Response function for the alpha spin channel, in 1/eV² |
| SP_xtbml_response_beta | Response function for the beta spin channel, in 1/eV² |
| SP_xtbml_ext_HOAO_alpha | Extended HOAO energy for the alpha spin channel, in eV |
| SP_xtbml_ext_HOAO_beta | Extended HOAO energy for the beta spin channel, in eV |
| SP_xtbml_ext_LUAO_alpha | Extended LUAO energy for the alpha spin channel, in eV |
| SP_xtbml_ext_LUAO_beta | Extended LUAO energy for the beta spin channel, in eV |
| SP_xtbml_ext_gap_alpha | Extended HOAO–LUAO energy gap for the alpha spin channel, in eV |
| SP_xtbml_ext_gap_beta | Extended HOAO–LUAO energy gap for the beta spin channel, in eV |
| SP_xtbml_ext_chem_pot_alpha | Extended chemical potential for the alpha spin channel, in eV |
| SP_xtbml_ext_chem_pot_beta | Extended chemical potential for the beta spin channel, in eV |
| **Density-based properties** | |
| SP_xtbml_q_A_alpha | Atomic partial charges for the alpha spin channel, in *e* |
| SP_xtbml_q_A_beta | Atomic partial charges for the beta spin channel, in *e* |
| SP_xtbml_dipm_A_alpha | Atomic dipole moment magnitude for the alpha spin channel, in *ea*_0_ |
| SP_xtbml_dipm_A_beta | Atomic dipole moment magnitude for the beta spin channel, in *ea*_0_ |
| SP_xtbml_dipm_s_alpha | s-shell dipole moment magnitude for the alpha spin channel, in *ea*_0_ |
| SP_xtbml_dipm_s_beta | s-shell dipole moment magnitude for the beta spin channel, in *ea*_0_ |
| SP_xtbml_dipm_p_alpha | p-shell dipole moment magnitude for the alpha spin channel, in *ea*_0_ |
| SP_xtbml_dipm_p_beta | p-shell dipole moment magnitude for the beta spin channel, in *ea*_0_ |
| SP_xtbml_dipm_d_alpha | d-shell dipole moment magnitude for the alpha spin channel, in *ea*_0_ |
| SP_xtbml_dipm_d_beta | d-shell dipole moment magnitude for the beta spin channel, in *ea*_0_ |
| SP_xtbml_qm_A_alpha | Atomic quadrupole moment magnitude for the alpha spin channel, in *ea*_0_² |
| SP_xtbml_qm_A_beta | Atomic quadrupole moment magnitude for the beta spin channel, in *ea*_0_² |
| SP_xtbml_qm_s_alpha | s-shell quadrupole moment magnitude for the alpha spin channel, in *ea*_0_² |
| SP_xtbml_qm_s_beta | s-shell quadrupole moment magnitude for the beta spin channel, in *ea*_0_² |
| SP_xtbml_qm_p_alpha | p-shell quadrupole moment magnitude for the alpha spin channel, in *ea*_0_² |
| SP_xtbml_qm_p_beta | p-shell quadrupole moment magnitude for the beta spin channel, in *ea*_0_² |
| SP_xtbml_qm_d_alpha | d-shell quadrupole moment magnitude for the alpha spin channel, in *ea*_0_² |
| SP_xtbml_qm_d_beta | d-shell quadrupole moment magnitude for the beta spin channel, in *ea*_0_² |
| SP_xtbml_p_s_alpha | Mulliken s-shell populations for the alpha spin channel |
| SP_xtbml_p_s_beta | Mulliken s-shell populations for the beta spin channel |
| SP_xtbml_p_p_alpha | Mulliken p-shell populations for the alpha spin channel |
| SP_xtbml_p_p_beta | Mulliken p-shell populations for the beta spin channel |
| SP_xtbml_p_d_alpha | Mulliken d-shell populations for the alpha spin channel |
| SP_xtbml_p_d_beta | Mulliken d-shell populations for the beta spin channel |

Table SI1: Atom-resolved geometry-, energy-, orbital energy-, density-based xTBML properties included in the ASE-native JSON representation. Quantities are reported separately for the *alpha* and *beta* spin channels. All listed columns are stored as JSON-encoded arrays under the *key_value_pairs* column.
